# C-reactive protein to lymphocyte ratio combined with clinical features to construct a predictive model for upper gastrointestinal bleeding due to peptic ulcer

**DOI:** 10.1016/j.clinsp.2025.100644

**Published:** 2025-04-23

**Authors:** Hong Song, Juan Yang, Jiao Li, Cui Deng, SiMin Zhang, Sheng Zheng

**Affiliations:** a Department of Gerontology, Taiyuan Central Hospital, Taiyuan City, Shanxi Province, China; b Department of Gastroenterology, Affiliated Hospital of Yunnan University, Kunming City, Yunnan Province, China; c Graduate School of Clinical Medicine, Dali University, Dali City, Yunnan Province, China; d Department of Gastroenterology, The Third People's Hospital of Yunnan Province, Kunming City, Yunnan Province, China

**Keywords:** Peptic ulcer, Upper gastrointestinal bleeding, C-reactive protein-to-lymphocyte ratio, Column chart, Prediction model

## Abstract

**Objective:**

This research aimed to determine the feasibility and accuracy of CLR and clinical features to formulate a prediction model for Peptic Ulcer (PU)-induced Upper Gastrointestinal Bleeding (UGIB).

**Methods:**

The clinical data of 146 PU patients were prospectively collected, and patients were divided into the UGIB group (*n* = 48) and the non-UGIB group (*n* = 98). The factors affecting UGIB were analyzed using multifactorial logistic regression and collinearity analysis. The prediction model of UGIB was constructed, the predictive value of which was analyzed using the Receiver Operating Characteristic Curve (ROC) and Area Under the Curve (AUC), while the accuracy was analyzed using the calibration curve and Hosmer Lemeshow goodness-of-fit tests, and the application value was assessed using decision curve analysis (DCA).

**Results:**

Statistical significance was observed between the two groups regarding HP infection, ulcer diameter, ulcer stage, use of nonsteroidal anti-inflammatory drugs, Neutrophil, LYM, NEUT/LYM Ratio (NLR), CRP, and CLR. HP infection, ulcer stage, use of NSAIDs, NLR, and CLR were independent risk factors for UGIB, and PCT was a non-independent risk factor. The AUC for this model was 0.921. The calibration curve of the model matched the actual curve. The model achieved a better fitting effect in predicting UGIB (χ^2^ = 8.5069, df = 8, *p* = 0.3856) and had a better clinical application value.

**Conclusion:**

A predictive model for PU-induced UGIB, based on CLR and clinical features, can assist in developing clinical treatment plans to prevent UGIB.

## Highlights

• CRP levels in patients with PU with UGIB were significantly higher than those without UGIB.
• PU patients with UGIB had a higher CLR compared to those without UGIB.
• HP infection is an independent risk factor for UGIB.

## Introduction

Peptic Ulcer (PU) is a common digestive disease caused mainly by the digestive action of gastric acid and pepsin on the gastric or duodenal mucosa.^1^ If left untreated, PU may penetrate the gastric or duodenal wall and cause serious complications. One of the most prevalent and severe complications is Upper Gastrointestinal Bleeding (UGIB), typically appearing as vomiting blood or having black stools, and in severe instances, it can result in hemorrhagic shock and pose a threat to life.^2^ Endoscopy is the main method for diagnosis and treatment of PU, and in recent years, some researchers have constructed prediction models based on endoscopic signs. Endoscopy, being an invasive procedure, must take into account the presence of cardiopulmonary disease, gastrointestinal perforation, shock, and other contraindications. Therefore, it is of great significance to search for a safe and accurate method to predict the risk of UGIB in patients with PU, in order to take timely interventions to improve the patient's prognosis.

C-Reactive Protein (CRP) is a reactive protein produced by the liver during the acute phase. Its levels in the serum increase quickly within hours when the body experiences infection, inflammation, or trauma, and the extent of this rise is related to the severity of the condition.^3^ In clinical practice, CRP is a widely recognized indicator of inflammation severity and is related to a range of gastrointestinal diseases.^4^, ^5^, ^6^ The immune system relies heavily on Lymphocytes (LYM), including T-cells, B-cells, and natural killer cells, to perform specific immune responses. These cells are capable of identifying and eliminating pathogens, tumor cells, and other invaders. During the inflammatory response, the number and function of LYM are altered accordingly, and changes in their counts can reflect the immune status of the body. CRP/Lymphocyte Ratio (CLR) has recently emerged as a novel biomarker with prognostic potential in a range of diseases such as pancreatic cancer,^7^ colorectal cancer,^8^ and COVID-19.^9^

UGIB significantly influences the prognosis of PU patients, and identifying high-risk patients early and intervening promptly can lower mortality rates. Studies have not yet looked into CLR's ability to predict UGIB in patients suffering from PU. Therefore, the aim of this study is to explore the clinical characteristics of patients with PU with UGIB and the influencing factors mainly of CLR, to establish a prediction model for predicting UGIB due to PU. This is intended to serve as a theoretical guide for clinical care to perform early assessments and screenings, adopt targeted actions to bolster nursing prevention and control, and effectively handle PU patients with UGIB risks.

## Materials and methods

### Patient data

The study involved 146 PU patients who were admitted to Taiyuan Central Hospital from March 2021 to March 2023. Inclusion criteria: 1) Meeting the diagnostic criteria of PU (10); 2) Age ≥18-years; 3) Complete relevant clinical data. Exclusion criteria: 1) Previous history of gastrointestinal surgery such as stomach, duodenum, etc.; 2) Combined with serious hematologic diseases; 3) Combined with other gastrointestinal diseases such as gastric cancer, gastrinoma, etc.; combined with cardiac, hepatic, renal, and other vital organ insufficiencies; and 4) Combined with psychiatric disorders, which could not be communicated normally. The study was approved by the Ethics Committee of Taiyuan Central Hospital, and the families signed an informed consent form.

### General data collection

Collected were medical records and follow-ups on test results. 1) General information was collected such as name, gender, age, use of NSAIDs, basic medical history (liver disease, heart failure), initial symptoms (vomiting blood, black stool, abdominal pain, abdominal distension, etc.), pulse, systolic blood pressure, and fainting. 2) During the hospital stay, data on endoscopic examination outcomes and treatment actions (such as blood transfusion, endoscopic procedures, and surgeries like interventional embolization and open surgery) were gathered. 3) Patients were divided into the group with complicated UGIB and the group without complicated UGIB.

Hypertension was assessed by either the use of antihypertensive drugs or having a diastolic blood pressure of 90 mmHg or greater and/or a systolic blood pressure of 160 mmHg or greater. Diabetes mellitus was diagnosed by using medications to manage blood glucose or throUGIB a glucose tolerance test, with 2-hour blood glucose levels at or above 11.1 mmoL/L and fasting blood glucose at or above 7.0 mmoL/L. UGIB diagnosis was based on symptoms like black stools, fatigue, melanomatosis, positive results for blood in gastric contents and fecal occult blood, a decrease in red blood cells and hemoglobin levels 3‒4 h after acute bleeding, and an increase in blood urea nitrogen levels.

### Biochemical indexes

Early in the morning of the next day of admission, 4 mL of fasting venous blood samples were collected from all patients with EDTA tubes and centrifuged at 3000 r/min with a radius of 8.5 cm for 10 min. The supernatant was taken and stored at −80 °C. CRP and Procalcitonin (PCT) were detected by using a BS-280 automatic biochemical analyzer (Mindray, Shenzhen, China) and supporting reagents. Serum Neutrophil (NEUT) count, LYM count, and platelet count were detected by BC-6800Plus automatic blood cell analyzer (Mindray). NLR = NEUT count/LYM count. CLR = CRP/LYM count, unit mg/109.

### Statistical analysis

Statistical analysis was performed using SPSS26.0. Data that followed a normal distribution were represented as mean ± standard deviation, and differences between groups were analyzed using the independent samples *t*-test. Data not following normal distribution were represented as median or interquartile range, and differences between groups were analyzed using the Mann-Whitney *U* test. Count data were shown as either percentages or frequencies, with the Chi-Square test employed to examine group differences. Factors influencing upper gastrointestinal bleeding were analyzed using stepwise backward logistic regression, and collinearity analysis was performed for these factors. Using the R4.2.3 software and rms program package, a prediction model for UGIB due to PU was developed. The model's discriminatory power was evaluated using ROC curves, its accuracy was assessed with calibration curves and Hosmer Lemeshow tests, and its clinical value was determined using DCA curves. Statistical significance was defined as *p* < 0.05 using a two-sided test.

## Results

### Clinical data of PU patients

Out of 146 patients with PU, 48 (32.88 %) also had UGIB and were classified as the UGIB group, while 98 patients (67.62 %) had uncomplicated UGIB and were classified as the non-UGIB group. Comparison of the baseline data between the two groups yielded that the differences between the two groups were not statistically significant (*p* < 0.05) between age, gender, history of smoking, alcoholism, history of hypertension, history of diabetes mellitus, location of the ulcer, duration of the disease, preference for stimulating foods, and WBC. The differences between HP infection, ulcer diameter, ulcer stage, and use of NSAIDs, NEUT, LYM, NLR, CRP, and CLR were statistically significant (*p* < 0.05) (Table 1).

**Table 1:** Combined UGH and clinical data of PU patients.

Variables	UIGB group (*n* = 48)	Non-UIGB group (*n* = 98)	p-value
Age	51.33 ± 5.54	50.29 ± 5.12	0.26
Gender			0.542
Male	29 (60.42 %)	54 (55.10 %)	
Female	19 (39.58 %)	44 (44.90 %)	
Smoking	17 (35.42 %)	31 (31.63 %)	0.709
Drinking	32 (64.58 %)	25 (68.37 %)	
HP infection			0.002
Yes	34 (70.83 %)	43 (43.88 %)	
No	14 (29.17 %)	55 (56.12 %)	
Hypertension	22 (45.83 %)	43 (43.88 %)	0.861
Diabetes mellitus	12 (25.00 %)	23 (23.47 %)	0.839
Location of ulcer			0.725
Gastric	27 (56.25 %)	51 (52.04 %)	
Duodenum	21 (43.75 %)	47 (47.96 %)	
Course of ulcer (years)	2.23 ± 1.21	2.18 ± 1.06	0.762
Ulcer diameter			<0.001
≤ 20 mm	13 (27.08 %)	59 (60.20 %)	
> 20 mm	35 (72.92 %)	39 (39.80 %)	
Ulcer stage			<0.001
Active	35 (72.92 %)	43 (43.88 %)	
Inactive	13 (27.08 %)	55 (56.12 %)	
Preference for stimulating foods	20 (41.67 %)	32 (32.65 %)	0.358
Use of non-steroidal anti-inflammatory drugs	22 (45.83 %)	19 (19.39 %)	<0.001
Laboratory indicators			
WBC count (× 109/L)	12.15 ± 2.14	11.76 ± 2.26	0.333
Neutrophil count (× 109/L)	8.02 (7.42, 8.83)	7.57 (6.76, 8.60)	0.049
Lymphocyte count (× 109/L)	2.59 (2.41, 2.84)	3.21 (2.97, 3.56)	<0.001
CRP (mg/L)	5.06 (4.78, 5.59)	4.98 (4.54, 5.26)	0.02
PCT (ng/mL)	3.51 ± 0.52	3.07 ± 0.49	<0.001
CLR	1.95 (1.80, 2.27)	1.54 (1.37, 1.72)	<0.001

### Multifactorial logistic regression analysis of complicated UGIB in PU patients

Using gender, age, and disease duration as correction variables, the statistically significant variables in the above clinical data were included in the stepwise backward multifactorial logistic regression analysis to derive that HP infection, ulcer stage, use of NSAIDs, NLR, and CLR were the independent risk factors for UGIB (*p* < 0.05), and PCT was a non-independent risk factor (Table 2).

**Table 2:** Multifactorial logistic regression analysis of complicated UGIB in PU patients.

Variables	β	S.E	*Z*	OR (95 % CI)	p
HP infection	1.44	0.73	1.96	4.21 (1.01‒17.70)	0.05
Ulcer stage	2.25	0.79	2.86	9.46 (2.02‒44.21)	0.004
Use of non-steroidal anti-inflammatory drugs	1.65	0.84	1.97	5.20 (1.01‒26.79)	0.049
NLR	2.54	0.81	3.14	12.74 (2.60‒62.41)	0.002
PCT	1.29	0.72	1.79	3.64 (0.89‒15.01)	0.073
CLR	3.44	1.42	2.42	21.32 (1.92‒510.27)	0.016

### Collinearity analysis of factors influencing the complication of UGIB in PU patients

HP infection, ulcer stage, use of NSAIDs, NLR, PCT, and CLR in multifactorial logistic regression analysis were subjected to collinearity analysis, and all tolerances exceeded 0.1, and all variance inflation factors were below 10, indicating no multicollinearity (Table 3).

**Table 3:** Collinearity analysis of factors affecting concomitant UGIB in PU patients.

Variables	T-value	p-value	Tolerance	VIF
HP infection	2.606	0.01	0.953	1.049
Ulcer stage	3.802	<0.001	0.959	1.043
Use of non-steroidal anti-inflammatory drugs	2.482	0.014	0.946	1.057
NLR	3.965	<0.001	0.568	1.76
PCT	3.292	0.001	0.902	1.108
CLR	3.678	<0.001	0.556	1.797

### Predictive value of logistic regression model for PU patients with concurrent UGIB

**Fig. 1:**
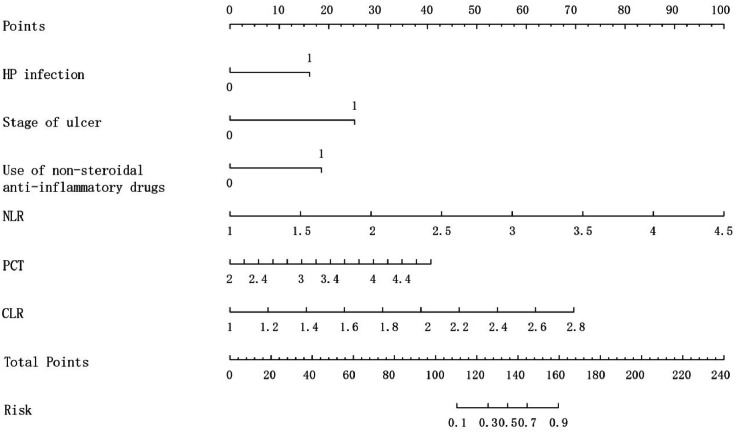
Predicted column line graph of concomitant UGIB in patients with PU.

The authors constructed a clinical prediction model based on the appeal of multifactorial logistic backward stepwise regression. For the convenience of clinical application, the model was visualized using a column-line diagram (Fig. 1). The column-line diagram consists of six factors: HP infection, ulcer stage, use of NSAIDs, NLR, PCT, and CLR, which are presented through the elements of scores, predictive probabilities, and line segments. Plotting a vertical line up to Points determines the scores for individual risk variables, multiple factor scores are summed to Total Points, and then a vertical line down to Risk determines the corresponding risk probability.

### Comparison of ROC and DCA curves for UGIB prediction by column line graphs

**Fig. 2:**
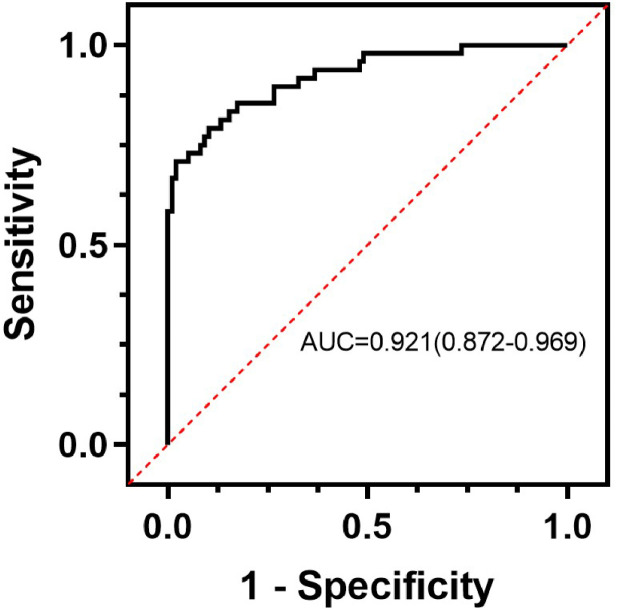
ROC curve of the predictive model for PU with UGIB.

**Fig. 3:**
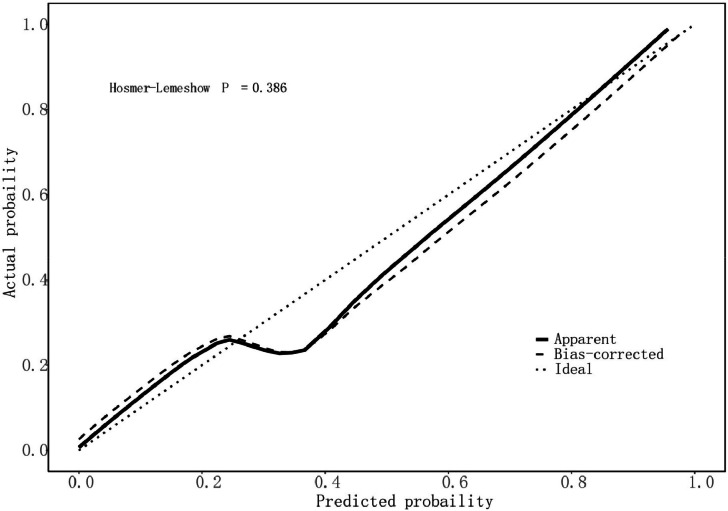
Calibration curves of the UGIB prediction model.

**Fig. 4:**
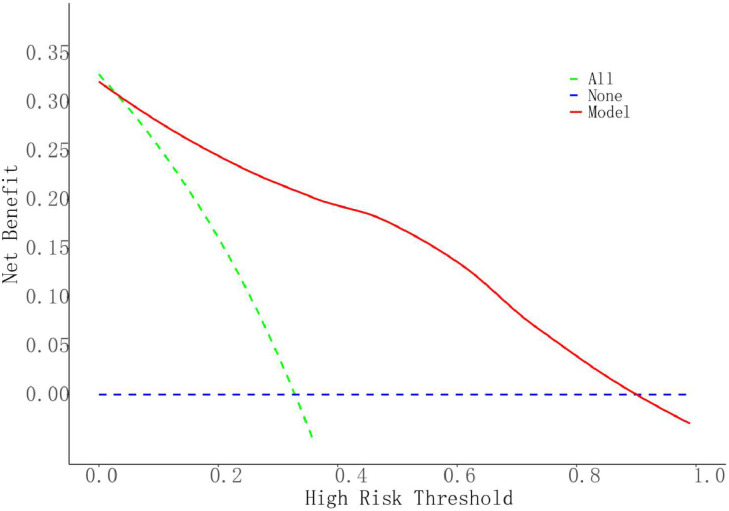
Evaluation of the clinical utility of the UGIB prediction model (DCA curve).

The ROC curve was used to evaluate the discrimination of the model and the AUC was calculated (Fig. 2). The AUC of the model was 0.921 (95 % CI 0.872‒0.969), which indicated that the model had a high predictive value. The goodness of fit was tested by the Hosmer-Lemeshow Test (HL Test), and the results showed χ^2^ = 8.5069, df = 8, *p* = 0.3856, indicating that the model fits well, and there is no statistically significant difference between the current model and the ideal model. The calibration curve is plotted to demonstrate the accuracy of the model (Fig. 3). The results showed good accuracy and consistency of the model. The clinical utility of the model in the dataset was assessed by plotting a DCA curve with the high-risk threshold probability as the horizontal coordinate and the vertical coordinate as the net benefit rate (Fig. 4), with the red solid line representing the predictive model, the green dashed line (All) representing the hypothesis that all patients with PU experience UGIB, and the blue dashed line (None) representing the hypothesis that all patients do not experience UGIB, which can be seen in the study model has a net benefit in all 0‒0.9 risk threshold probability scenarios, thus the model has good clinical application.

## Discussion

PU is a common gastrointestinal disease. When the defense mechanism of the gastric mucosa is damaged, it is susceptible to attack by harmful substances such as gastric acid and pepsin, which injure the submucosal blood vessels and lead to blood spillage, thus causing UGIB.^10^ Symptoms of UGIB include vomiting blood and black stools, which may be life-threatening in severe cases and require prompt intervention and treatment.^11^ There have been many in-depth studies on the occurrence and development of PU, and it is believed that the interference of inflammation plays an important role in disease progression. However, in predicting the risk factors for PU complicating UGIB, these studies have mostly used conventional inflammatory indicators for analysis or simple comparisons of biomarkers related to inflammatory response, and the dynamics of their changes during treatment are not clear.^12^ Thus, creating a predictive model that effectively combines serum markers and clinical features is crucial for preventing and treating PU with UGIB due to its high diagnostic efficacy and compliance. In this study, the authors assessed the risk factors for UGIB in patients with PU and constructed a risk model for predicting UGIB. The model demonstrated strong accuracy and was clinically applicable. The findings could enhance early detection of high-risk patient groups and encourage the proactive implementation of suitable clinical interventions, thus improving patient outcomes.

CRP is an acute-phase reactive protein whose serum levels are elevated during inflammation.^13^ CRP levels are detected to be elevated in patients with gastrointestinal bleeding.^14^^,^^15^ Similar to these studies, the present study found that CRP levels in patients with PU with UGIB were significantly higher than those without UGIB. However, CRP only reflects the presence and degree of inflammation and does not reflect changes in the immune status of the body. The CLR metric is a combination of CRP and LYM count. LYMs are vital to the immune system, and variations in their count can reveal the status of the body's immune health. Therefore, CLR not only reflects the degree of inflammatory response but also changes in immune function. For example, in infectious diseases, an increase in CRP indicates the presence of inflammation, but when combined with the CLR calculated from the LYM count, a decrease in the LYM count is associated with a more pronounced increase in the CLR, indicating that the immune function of the body may be suppressed in an inflammatory state, which can help to assess the condition more comprehensively.^9^^,^^16^ Several studies have shown that elevated CLR is an independent risk factor for poor prognosis in diseases, including cancers and inflammatory diseases.^9^^,^^17^^,^^18^ Logistic regression analysis in this study showed that PU patients with UGIB had a higher CLR compared to those without UGIB. The presence of UGIB with PU causes stress in the body, initiating an inflammatory response. On the one hand, inflammatory mediators such as cytokines released from damaged tissues stimulate the liver to synthesize CRP, leading to an increase in its level. Bleeding, on the other hand, can lead to immune regulation disorders, impacting the distribution and function of LYM and causing changes in their numbers. This imbalance between inflammation and immunity causes changes in the CLR.

HP infection destroys the gastric and duodenal mucosa and is the main cause of PU.^19^ The absence of typical epigastric pain in many PU patients often prevents early diagnosis and delays treatment, increasing the risk of bleeding.^20^ HP infection triggers the release of inflammatory mediators, leading to elevated serum PCT and CRP levels.^21^^,^^22^ In the present study, HP infection accounted for 70.83 % of patients with UGIB, and logistic regression results showed that it is an independent risk factor for UGIB. About 25 % of patients taking NSAIDs for a long period of time develop PU, and some of them also develop complications such as PU bleeding.^23^ In the prediction model, taking NSAIDs was likewise an independent risk factor for UGIB. Active ulcers are usually accompanied by a more intense inflammatory response, increased tissue fragility, and immature neovascularization prone to rupture, which further increases the risk of severe bleeding. Therefore, the results of this analysis showed that the ulcer stage as active is also an influential factor for UGIB in patients with PU.

In this study, a prediction model was constructed by analyzing the above clinical characteristics of the patients: logit(p)=1.44×HPinfection+2.25×ulcerstage+1.65×useofNSAIDs+2.54×NLR+1.29×PCT+3.44×CLR−20.59 A column-line graph was plotted based on the modified model. The AUC for the column chart was 0.921 (95 % CI 0.872‒0.969). Calibration curve analysis showed that the calibration curve of the model for predicting UGIB matched with the actual curve, and after the Hosmer-Lemeshow test, it was concluded that the model predicted UGIB with a good fitting effect (χ^2^ = 8.5069, df = 8, *p* = 0.3856), and the DCA curve showed that the model had good clinical application value.

This research was conducted with a small sample size at a single center, and since the participants were from one hospital, the findings may not be applicable to hospitals at different levels. It is also unclear if this prediction model can be used for other groups. Therefore, multi-center, large-sample studies are needed for further validation.

In conclusion, the prediction model constructed by CLR in combination with clinical characteristics has important predictive value for UGIB caused by PU, which can help the clinic to identify high-risk patients at an early stage, take targeted interventions, and improve the prognosis of patients.

## Availability of data and materials

The datasets used and/or analyzed during the present study are available from the corresponding author on reasonable request.

## Ethics approval

The present study was approved by the Ethics Committee of Taiyuan Central Hospital and written informed consent was provided by all patients prior to the study start. All procedures were performed in accordance with the ethical standards of the Institutional Review Board and The Declaration of Helsinki, and its later amendments or comparable ethical standards.

## Funding

1. Yunnan Health Training Project of High-Level Talents (nº d-2,024,049).
2. Yunnan Province High-level Scientific and Technological Talents and Innovation Team Selection Special ‒ Young and Middle-aged Academic and Technical Leaders Reserve Talent Project (nº 202405AC350067).
3. Basic Research Joint Special General Project of Yunnan Provincial Local Universities (part) (nº 202301BA070001–029).
4. The 8th Research Project of Education and Teaching Reform of Dali University (Special Medical Education Reform Project, nº 2022JGYX08–01).
5. Basic Research Joint Special General Project of Yunnan Provincial Local Universities (part) (nº 202301BA070001–044).
6. Scientific Research Fund Project of Education Department of Yunnan Province (nº 2023J0926).
7. The 8th Research Project of Education and Teaching Reform of Dali University (Special Medical Education Reform Project, nº 2022JGYX08–02).

## Declaration of competing interest

The authors declare no conflicts of interest.
